# Scale-Free Neurodynamics as Functional Fingerprint of Brain Regions

**DOI:** 10.3390/bioengineering13030323

**Published:** 2026-03-11

**Authors:** Karolina Armonaite, Franca Tecchio, Baingio Pinna, Camillo Porcaro, Livio Conti

**Affiliations:** 1 Faculty of Mathematics and Natural Sciences, Kaunas University of Technology, 44249 Kaunas, Lithuania; karolina.armonaite@ktu.lt; 2 Laboratory of Electrophysiology for Translational Neuroscience, Institute of Cognitive Sciences and Technologies—Consiglio Nazionale Delle Ricerche, 00196 Rome, Italy; francamatilde.tecchio@cnr.it (F.T.); camillo.porcaro@unipd.it (C.P.); 3 INFN—Istituto Nazionale di Fisica Nucleare, Sezione Roma Tor Vergata, 00133 Rome, Italy; 4 Department of Biomedical Science, University of Sassari, 07100 Sassari, Italy; baingio@uniss.it; 5 Biomedical Engineering Research to Advance and Innovate Translational Neuroscience (BRAIN Unit), Department of Neuroscience & Padova Neuroscience Center, University of Padova, 35128 Padova, Italy; 6 Faculty of Engineering, Uninettuno University, 00186 Rome, Italy; 7 IAPS—INAF Istituto di Astrofisica e Planetologia Spaziali—Istituto Nazionale di Astrofisica, 00133 Rome, Italy

**Keywords:** scale-free dynamics, multifractal, power-law, neurodynamics, resting-state activity, brain functional parcellation

## Abstract

This study investigates the ongoing electrical activity of local neural networks—referred to as neurodynamics—across 37 anatomically defined brain regions. We analyzed stereotactic intracranial EEG (sEEG) recordings from 106 subjects during wakeful rest, focusing on scale-free (power-law) properties to determine whether distinct brain regions exhibit unique neurodynamic signatures. Results revealed a power-law regime in two frequency ranges (approximately 0.5–4 Hz and 33–80 Hz). Notably, the power-law exponent (slope) in the high-frequency band differed significantly between cortical and subcortical areas (*p* < 0.01). These findings suggest that local neurodynamics, as reflected in scale-free characteristics, may serve as a functional “fingerprint” for brain region classification. This approach may contribute to functional brain parcellation efforts and offer new insights into the intrinsic organization of neuronal networks as revealed by resting-state activity analysis.

## 1. Introduction

The ongoing electrical activity within the brain network, known as neurodynamics, reflects the structure and function of the neuronal populations responsible for its generation. When detected through stereotactic intracranial EEG (sEEG) recordings, this activity often displays a non-periodic, fractal-like pattern emerging from the interplay between neuronal excitatory (E) and inhibitory (I) connections. Previous evidence suggests that the local neurodynamics of cortical regions, even in resting-state conditions, may exhibit a particular behavior that could be considered a functional signature of each area [1,2]. Various linear and non-linear measures have been employed to assess the specific activity across different brain regions. Cortical areas have been shown to express distinct frequency band activity in the power spectral density (PSD) function [1,3,4]. Some authors also found that PSDs can be automatically clustered using machine learning techniques [2,4]. A promising approach for this classification has involved fractal analysis [5], where researchers applied Higuchi’s fractal dimension to characterize the complexity of neurodynamics across distinct brain areas [6,7,8]. However, evaluating and comparing such measures can be challenging, as many depend on parameters that are not always consistently controlled [9].

An emerging and compelling line of research has focused on the scale-free (1/*f^β^*) component of the brain’s electrical signal, characterized by the power-law exponent *β*, which reflects the non-oscillatory background activity underlying neural population dynamics. This component, often masked by narrowband oscillations, captures the aperiodic structure of the PSD and provides valuable insight into the intrinsic dynamical state of the brain [10,11]. Specifically, the slope and intercept of the 1/*f^β^* component have been associated with the excitation/inhibition (E/I) balance, arousal, and cognitive state [12,13,14]. Furthermore, it has been argued that the scale-free 1/*f^β^* structure in EEG emerges from biophysical synaptic dynamics and constitutes an essential, state-dependent component of neural activity that constrains the interpretation of oscillatory phenomena [15].

Donoghue et al. [16] introduced a principled method for parameterizing neural power spectra into periodic and aperiodic components, enabling a more accurate interpretation of underlying neural processes. Voytek et al. [17] further demonstrated that age-related changes in the spectral slope reflect meaningful alterations in cortical circuitry, while distinct developmental changes in aperiodic dynamics during infancy have also been reported [18,19]. Gerster et al. [20,21,22] further emphasized methodological challenges in separating these spectral components, offering guidelines to enhance reliability and interpretability. Unlike traditional narrowband metrics, which are sensitive to task-specific fluctuations and noise, 1/*f^β^* characteristics offer a more stable and generalizable measure of neural activity. This makes them a promising candidate for identifying intrinsic signatures of local neurodynamics.

Fractal-based techniques, such as Higuchi’s fractal dimension (HFD), have also been explored to quantify signal complexity and regional differentiation [4,6]. However, many of these methods require sensitive parameter tuning and lack a standardized physiological interpretation [23]. In contrast, the exponent and the intercept of 1/*f^β^* power-law behavior can be derived using well-established fitting techniques like least-squares regression [5,24], enabling a more robust and interpretable framework for measuring neural complexity and organization. Notably, intracranial electroencephalography is particularly sensitive to neural activity. Simultaneous electrocorticography (ECoG) and functional magnetic resonance imaging (fMRI) recordings during motor tasks have demonstrated that high-gamma power is the only frequency band in which electrophysiological responses consistently co-localize with fMRI activation across subjects [25,26]. Moreover, it was shown that the estimation of the sEEG power-law exponent in the high-frequency band is relatively insensitive to minor parameter changes yet effectively differentiates between cortical regions [27]. Together, these findings motivate the use of the high-frequency sEEG power-law exponent as a region-specific marker of local neurodynamics.

Building on this foundation, the present study investigates whether local neurodynamics, characterized by power-law scaling behavior, exhibits region-specific signatures across anatomically defined brain areas. By applying spectral analysis to sEEG recordings from physiologically healthy brain regions during resting wakefulness, we aim to determine whether distinct brain parcels display unique scale-free patterns, particularly in the high-frequency band (approximately 33–80 Hz). If successful, this approach could significantly advance our understanding of functional specialization in the cortex, aid in developing biomarkers for neuropsychiatric conditions, and support the creation of more interpretable and biologically grounded models for classifying brain activity using machine learning.

## 2. Materials and Methods

### 2.1. Dataset

**Figure 1:**
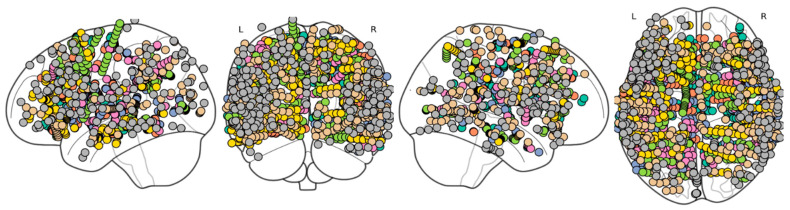
***sEEG channel positions***. *Representation of sEEG channels on the brain: left lateral, posterior, right lateral, and superior views*.

In this study, we analyzed the stereotactic-intracranial EEG (sEEG) recordings from 37 anatomically defined brain regions, using data from the Montreal Neurological Institute (MNI) dataset, available at https://mni-open-ieegatlas.research.mcgill.ca/ (accessed on 15 January 2026). The recordings were collected during wakefulness at rest from 106 subjects (age 33.1 ± 10.8 years, 54 males), yielding a total of 1772 channels, which were diversely distributed across regions (Figure 1). Even though subjects suffered from drug-resistant epilepsy, data provided by MNI and included in the study are only from brain areas not affected by the disease. This ensures that the analyzed signals reflect physiologically healthy activity. Recordings were obtained at least 72 h after stereotactic electrode implantation or one week after placement of subdural grids or strips, at a time when antiepileptic medications had not yet been reduced [1]. All recordings were 60 s long, sampled at 200 Hz [1,28,29,30].

**Figure 2:**
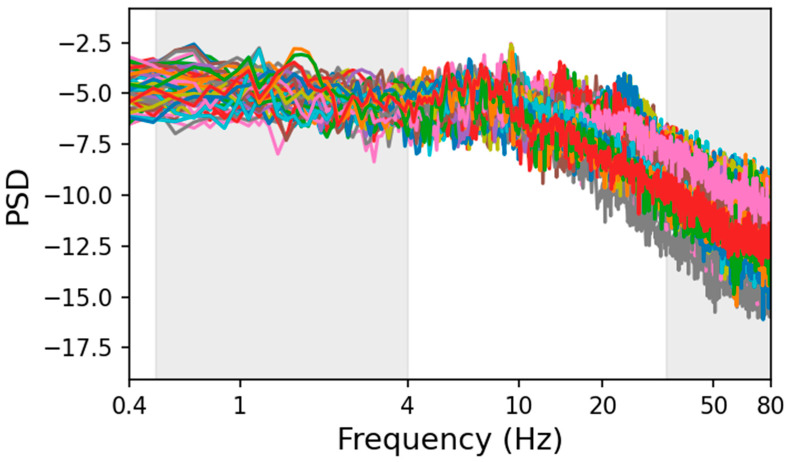
***Example of PSD from sEEG recordings.** The PSDs of the postcentral gyrus are plotted against frequency on a log-log scale. Different colors correspond to different channels. The grey shaded areas highlight the low-frequency (approx. 0.5–4 Hz) and high-frequency (approx. 34–80 Hz) bands where the PSD exhibits a power-law regime*.

The aim of this analysis was to investigate the existence of power-law regime in the PSD of sEEG recordings, possibly multiple power-law behaviors with different slopes in different frequency bands (Figure 2), and to determine whether neurodynamics in each brain region preserve distinctive features in the power-law exponents that could serve as a functional fingerprint of that area.

### 2.2. Scale-Free Properties of Gaussian and Brownian Noise

**Figure 3:**
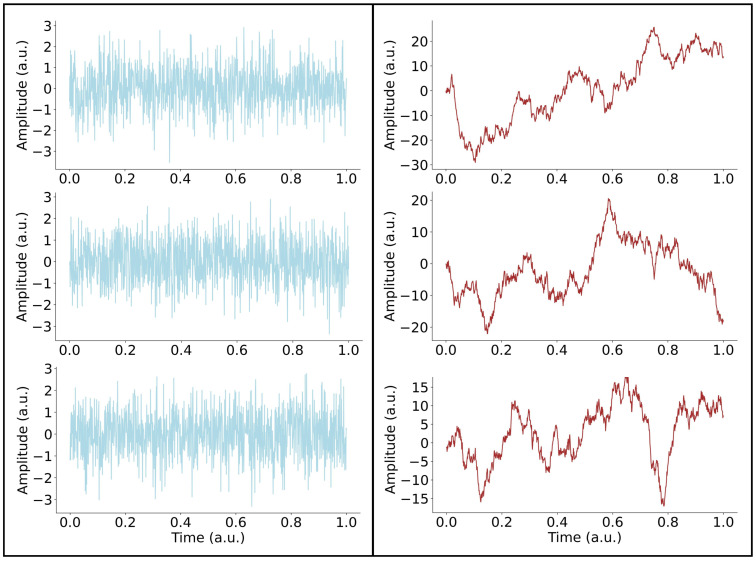
***Examples of white and Brownian noise.** On the left, we present three examples of white noise, which is stationary with a normal distribution. On the right, three examples of Brownian noise, which is a nonstationary signal characterized by a time-dependent variance*.

**Figure 4:**
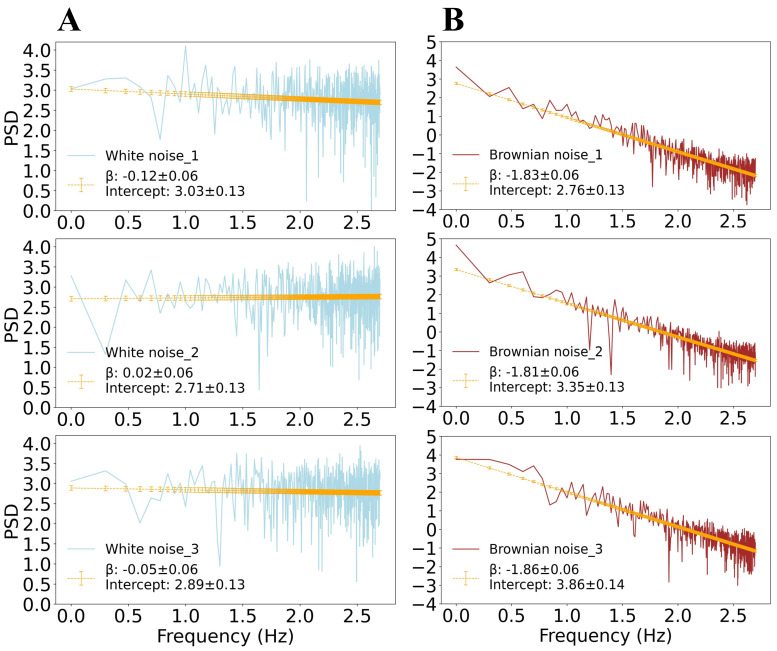
***Gaussian and Brownian noise power-law trends.** (**A**). The PSD of the three white noise segments from Figure 3, shown on a log-log scale, together with the linear fit (orange line) calculated using the least squares method. In the legend, the values of the slope (with |β| ≈ 0 for all cases) and intercept are provided. (**B**). PSD on a log-log scale for the three generated Brownian noise series from Figure 3. Panels show a power-law behavior with a constant slope over the entire frequency range. The orange line represents the fit obtained with the least-squares method and the error bars represent the fitting error; as in panel (**A**), the legend provides the fitting parameters*.

The power-law behavior of the PSD is a key feature of various signals such as Gaussian noise described by the normal probability distribution function:

$$W\left(t\right)=\frac{1}{\sqrt{2\pi \sigma }}e^{-\frac{{\left(t-\mu \right)}^{2}}{2\sigma^{2}}}$$

with mean *μ* and standard deviation *σ* (see examples in the left panels of Figure 3), and Brownian noise. The latter is defined as the limit of the sum of *W_i_* independent and identically distributed variables with $E\left[W_{i}\right]$ = 0 and $E\left[W_{i}^{2}\right]=\sigma^{2}$, defined as:

$$B\left(t\right)=\underset{n\to \infty }{\lim}\frac{1}{\sqrt{n}}\sum_{i=1}^{\left\lfloor nt\right\rfloor }W_{i}$$

where ⌊nt⌋ denotes the integer part of *nt* (see examples in the right panels of Figure 3). Figure 4 displays the PSD vs. frequency in log-log scale for three examples of simulated Gaussian (light blue, panels A) and Brownian (red, panels B) noise. Each PSD exhibits a power-law behavior *PSD*(*f*) = 1/*f^β^* with a constant slope over the entire frequency range. The orange line represents the linear fit computed using the least-squares method for estimating the values of slope and intercept as provided in the legend.

We observe that the spectrum of white noise is flat, with power-law exponent |*β*| ≈ 0 (Figure 4A). The Brownian noise exhibits a single power-law behavior across all frequency ranges with a power-law index |*β*| = 1.83 ± 0.06 (Figure 4B).

### 2.3. Removing Oscillatory Components from sEEG Signals

The plot of the PSD of sEEG signals vs. frequency on a double-logarithmic scale exhibits distinct portions of power-law behavior with different slopes as shown in Figure 2. Since sEEG signals contain prominent oscillatory activity peaks (alpha, beta, etc.), we further processed the recordings to reduce as much as possible the impact of periodic oscillations when evaluating the power-law exponent.

To attenuate alpha-band oscillations before estimating the scale-free parameters, we applied Butterworth filters tailored to two frequency ranges. Specifically, a 7th-order low-pass Butterworth filter, with an 8 Hz cutoff was used to isolate the low-frequency range (0.5–4 Hz), and a 7th-order high-pass Butterworth filter, with a 33 Hz cutoff was applied to isolate the high-frequency range (34–80 Hz). The Butterworth filter frequency response is defined as:

$$\left|H(n,j\omega )\right|=\frac{1}{\sqrt{1+{\left(\frac{\omega }{\omega_{c}}\right)}^{\pm 2n}}}$$

where *n* is the order of the filter and *ω_c_* is the cutoff frequency. This filtering approach aimed to minimize spectral distortions introduced by prominent alpha peaks (8–12 Hz), which have been observed to artificially bias the 1/*f* slope. This approach follows previous studies that have identified separate oscillatory and non-oscillatory components in neurophysiological signals [27,31].

### 2.4. Power-Law Behavior Evaluation of sEEG Across Brain Parcels

**Figure 5:**
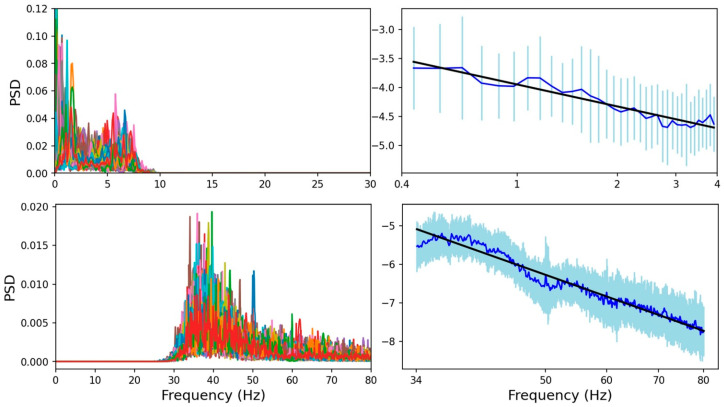
***Example of power-law behavior and fit for the case of postcentral gyrus.** Top panel: PSDs of channels in the postcentral gyrus plotted against frequency after low-pass filtering. On the left, all channels are plotted in distinct colors on a linear scale; on the right the average (blue) with the standard deviation (cyan) is shown on a log-log scale. The linear fit is overlaid in black. Bottom panel: The PSDs after high-pass filtering plotted against frequency are shown for each channel on a linear scale (left) and on average on a log-log scale (right) with the linear fit overlaid in black*.

For each brain region, we applied a filtering procedure to each sEEG channel to obtain low-pass and high-pass filtered signals. Subsequently, we estimated the PSD for each low- and high-pass filtered sEEG channel (segment duration: ~10 s), using Welch’s method, with a Hamming window and 2048-point FFT. Then, for each region separately, in both the low- and high-frequency ranges, we averaged the PSDs and evaluated the standard deviation across all subjects and channels. Since the variability between the subjects is higher than the within-subject one across channels [20], we adopted the most conservative approach and used the global standard deviation across all channels and subjects as the uncertainty estimate. The power-law behavior was evaluated across 37 brain regions for both the low- and high-frequency ranges separately, by log-transforming the frequencies and mean PSD values. To estimate the linear trend, specifically the slope (*β*), the weighted least-squares method was used. To account for uncertainty, the standard deviation was included as a weight during the fitting procedure: for each ordinate value $y_{i}=\log\left(PSD\left(f_{i}\right)\right)$ the weight is defined as $w_{i}=1/∆y_{i}^{2}$ with $∆y_{i}$ representing the uncertainty in $y_{i}$, that is the standard deviation (*σ_i_*) across the population. In Figure 5, the top panel illustrates the PSDs of all signals after low-pass filtering versus frequency on a linear scale (left) and the global average (with its standard deviation) versus frequency on a log-log scale together with the linear fit for *β* estimation (right). The bottom panel shows the same for the high-frequency range.

### 2.5. Tunable Parameters in Power-Law Fitting

The linear fitting of the PSD function involved several tunable parameters, including spectral resolution, low- and high-pass cutoff frequencies, and filter order. To assess the robustness of the results, multiple parameter configurations were systematically tested [20]. Specifically, we tested cutoff frequencies of 4 Hz and 8 Hz for the low-pass Butterworth filter, and 33 Hz and 50 Hz for the high-pass filter, as well as filter orders of *n* = 5 and *n* = 7 in both filters. In addition, spectral resolution was varied by adjusting the FFT window length (256, 1024, and 2048 samples). No statistically significant differences in the *β* exponent were observed across different parameter configurations. Therefore, we adopted the following conservative parameter set: 2048-point FFT, *n* = 7 filter order, low-pass cutoff at 8 Hz, and high-pass cutoff at 33 Hz. All signals were processed with the same procedure across regions; a grand average was then taken within each region, retaining the standard deviation of the fit.

### 2.6. Statistical Analysis

We performed an analysis of variance (ANOVA) to determine whether *β* values differ across the 37 brain regions, conducting separate analyses for the low- and high-frequency ranges. Post hoc pairwise comparisons were performed using Tukey HSD, with correction for multiple comparisons. Differences were considered statistically significant at *p* < 0.05.

Furthermore, we calculated the mean PSD value in the high-frequency band (33–80 Hz, γ frequency band) denoted as 〈PSD(f)〉_γ_ and computed Pearson’s *r* linear correlation coefficient between *β* and 〈PSD(f)〉_γ_ across the investigated brain areas with the aim to verify whether the slope is linearly dependent on the mean gamma band power.

## 3. Results

**Figure 6:**
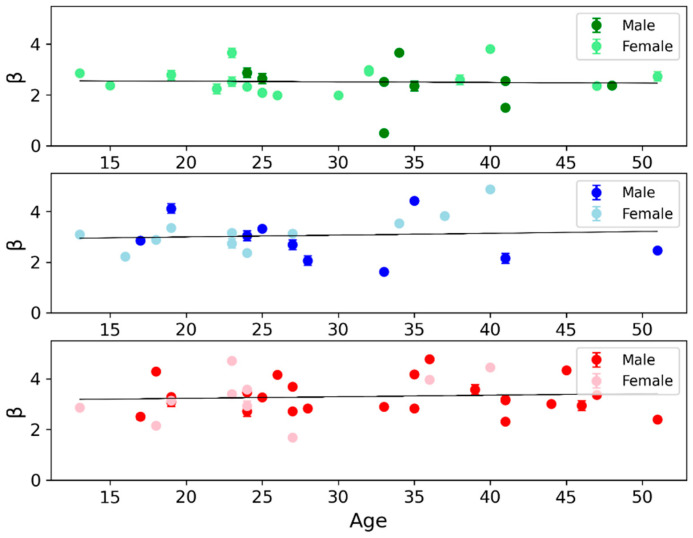
***Scatter plots of the power-law exponent β as a function of age and sex.** Example of scatter plots of individual β (that is estimated for each subject in the high-frequency range) as a function of age and separated by sex (male: darker markers; female: lighter markers) are shown for three brain regions: superior temporal (green), postcentral (blue), and precentral gyrus (red). For each subject, the error bar indicates uncertainty within the subject (that is the standard deviation evaluated across channels of the subject). In each region, the solid black line represents a linear regression fit across age. We can observe that β shows: (i) a substantial inter-individual variability, greater than that within a subject; (ii) a weak and non-significant age-related trend; and (iii) partially overlapping distributions between males and females*.

By plotting the PSD as a function of frequency, for all channels, separately in each of the 37 investigated brain regions, we observed two distinct linear trends, representing power-law regimes indicative of scale-free activity in the low (0.5–4 Hz) and high (34–80 Hz) frequency ranges (see Figure 2 for an example). As mentioned, and confirming literature reports [27], the inter-subject variability of *β* is higher than the intra-subject one in each investigated brain parcel, both in the low- and high-frequency ranges. This can be appreciated from the examples of scatter plots shown for three regions in Figure 6, where the individual *β* (estimated independently for each subject, in the high-frequency range) plotted against subjects’ age shows that the *β* error bars (due to the variability of channels within a subject) are smaller than the dispersion of *β* values across subjects. This supports our conservative approach of evaluating the global average of PSD and its standard deviation across all subjects and channels, and using this larger error in the weighted least-squares linear fit.

### 3.1. Regional Differences in β Calculated from High-Frequency Range

**Figure 7:**
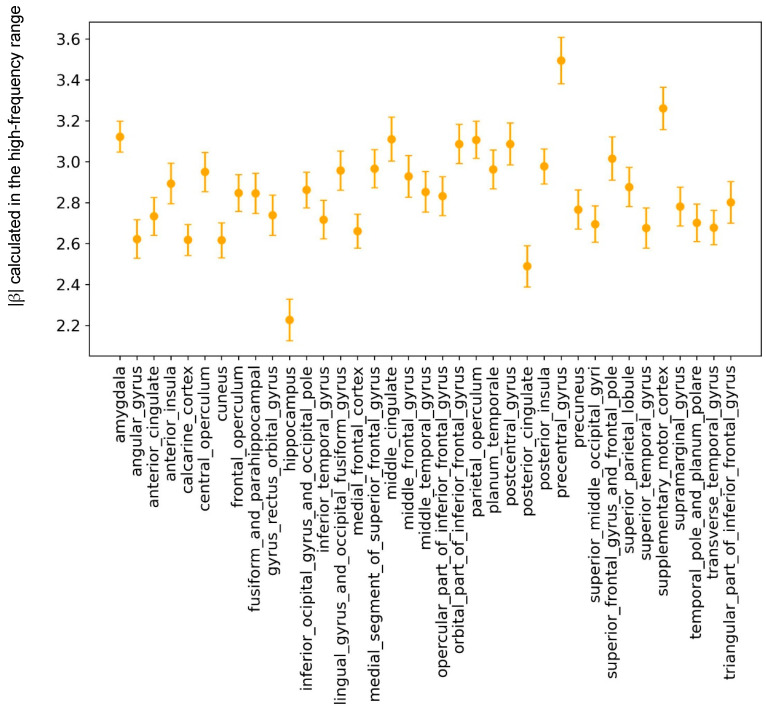
***Scatter plot of β values for the 37 investigated brain regions.** The data show the β value for each region estimated in the high-frequency range (33–80 Hz) with error bars representing the fitting error*.

**Figure 8:**
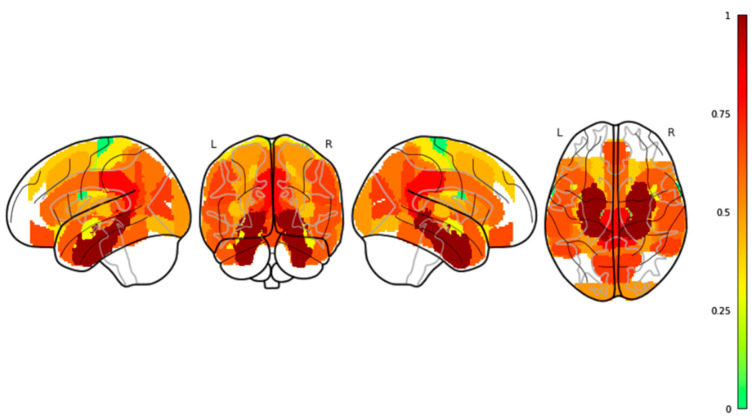
***Map of β values across regions on four spatial brain projections.** The investigated areas are colored based on the value of the ratio ${\Delta \beta }_{i}=\frac{\left(\beta_{i}-\beta_{min}\right)}{\left(\beta_{max}-\beta_{min}\right)}$, where $\beta_{i}$ is the β value for the i-th brain region, and β_min_ (β_max_) is the minimum (maximum) β value across regions. The ratio values range between 0 and 1*.

We found no significant effect of Region in the low-frequency range. However, we observed a significant main effect of Region in the high-frequency range (F_35,70_ = 315.82, *p* ≤ 0.0001). Significant differences were found in the high-frequency range between the precentral, postcentral, and superior temporal gyri in the cortex, and between the amygdala, hippocampus, and insula in the subcortical regions. These differences are illustrated in Figure 7 and Figure 8.

### 3.2. Investigating the Possible Dependence Between Gamma Spectral Power and Scale-Free Behavior

**Figure 9:**
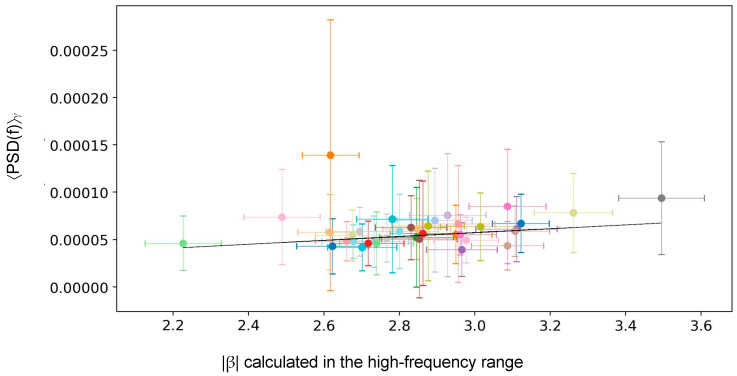
***Scatter plot across brain areas of gamma PSD vs. power-law exponent.** Scatter plot, across the 37 investigated areas, of 〈PSD(f)〉_γ_ (the normalized PSD averaged over the gamma band) vs. the power-law exponent β (estimated in the high-frequency range, 33–80 Hz). Different colors correspond to different brain areas. The black line indicates the linear fit aimed at studying the possible correlation between the gamma band PSD mean and the power-law exponent*.

We investigated the possible correlation between the normalized PSD averaged over the gamma band, denoted as 〈PSD(f)〉_γ_, and the power-law exponent *β* estimated in the high-frequency range (33–80 Hz). Figure 9 displays the scatter plot of 〈PSD(f)〉_γ_ vs. *β* across the 37 investigated areas. Notably, we observed a low dispersion of the mean PSD in the gamma band contrasted with a high dispersion of *β* slope values across regions. By applying a linear fit (black line) in Figure 9, we did not observe any significant correlation (r = 0.23, *p* = 0.17), suggesting a lack of dependence between the two quantities.

## 4. Discussion

Building on previous studies regarding the scale-free behavior of sEEG recordings [15,31,32] and specifically on the results of [27], which showed the possibility of distinguishing some cortical areas on the basis of the power-law exponent, in the present study we extended the investigation to 37 brain areas in order to test the hypothesis that the scale-free (1/*f*) characteristics of sEEG signals at rest can distinguish a larger number of brain regions. The PSD in both the low-frequency (0.5–4 Hz) and high-frequency (33–80 Hz) ranges exhibited a scale-free trend (with different slopes), consistent with previous reports, particularly in cortical areas [15,31,32]. Notably, as in [27], only in the high-frequency range did the power-law exponent *β* show a statistically significant variability between areas, suggesting that (while scale-free behavior is present also in the low-frequency range [33]) its spatial differentiation reaches statistical significance at higher frequencies.

One possible explanation for the lack of regional specificity of *β* values estimated in the low-frequency range across brain areas is the dominance of large-amplitude delta activity, which may obscure subtle variations in the aperiodic (scale-free) component. This is consistent with earlier observations that spectral content at low frequencies could mask underlying structural or functional distinctions in the aperiodic background [11,17]. Importantly, this does not imply that scale-free dynamics are absent in low frequencies, but rather that their variability, within the estimation errors of b, may not capture regionally distinct neurophysiological properties under resting-state conditions.

In contrast, the high-frequency slope demonstrated robust differences across both cortical and subcortical regions. This suggests that the power-law exponent *β* may reflect stable, intrinsic characteristics of local circuit organization, such as synaptic density or recurrent connectivity patterns. However, we also observed that the *β* slope was not significantly correlated with gamma-band power, suggesting that the aperiodic component reflects aspects of neural activity, distinct from those captured by traditional oscillatory measures.

While previous studies have interpreted the 1/*f* slope as a proxy for cortical excitability or E/I balance [14,16,17,34], such conclusions typically rely on task-based paradigms, developmental comparisons, or direct behavioral associations. Conversely, our study focused solely on resting-state sEEG data. As such, any interpretation of the *β* slope as a marker of excitability must be considered speculative without corroborating evidence from stimulation, behavior, or pathology. Moreover, other studies have shown that different EEG-derived markers of excitability may diverge, and not all aperiodic estimates track cortical excitability in the same way [35,36].

Nevertheless, the aperiodic EEG component has been shown to be useful in clinical settings. For instance, it serves as an electrical biomarker in distinguishing Alzheimer’s disease and healthy control subjects [37,38]. It was also found that such non-oscillatory neural activity measures are promising in detecting obsessive–compulsive disorder and depression [39,40,41]. Importantly, an altered 1/*f* slope could indicate a pathological excitatory/inhibitory imbalance, which could help to predict epileptic seizures [34,42].

It is also important to note that several known factors—such as recording conditions, age, medication, arousal state, and even hormonal cycle in women—could influence the 1/*f* slope [13,17,20]. In fact, it was shown previously that aperiodic-adjusted components in the theta range showed the strongest sensitivity to experimental condition, age, and their interaction. However, canonical power and isolated aperiodic components alone were less sensitive to condition differences [19,22,43]. Moreover, female sex steroid hormones across the menstrual cycle and during hormonal contraceptive use have been shown to modulate neurotransmitter systems and cortical excitability; nevertheless, recent evidence indicates that resting-state EEG alpha power and peak frequency as well as 1/*f* slope parameters may not differ significantly across menstrual phases or contraceptive status [44]. Consistent with [27], our results across all 37 brain areas show that the dependence of *β* on age is not statistically significant, or within the other sources of uncertainty, and the distributions of *β* values for males (darker markers) and females (lighter markers) are partially overlapping (see Figure 6 for an example).

Furthermore, future studies should incorporate detailed metadata on clinical factors, including female hormonal status, to improve the interpretability of aperiodic signal features and to investigate subtle modulatory effects that could contribute to inter-individual variability.

More broadly, the observed regional differences in *β* support the hypothesis that local neurodynamics may exhibit region-specific spectral fingerprints, as proposed in previous work on both aperiodic features and fractal-based complexity measures [4,6,7,8,45,46,47]. However, we emphasize that the presence of statistically significant differences does not, in itself, imply functional or behavioral relevance. Additional research is needed to establish whether these spectral distinctions map onto anatomical hierarchies, connectivity patterns, or functional roles.

Finally, while our findings contribute to the ongoing effort to characterize the aperiodic structure of brain activity, we acknowledge that this work builds upon, rather than introduces, a novel methodological approach. Foundational studies such as those by [16,17] have already demonstrated the utility and interpretability of separating periodic and aperiodic components in neural signals. Our contribution lies in applying these concepts to a stereotactic EEG dataset to assess regional specificity under well-defined resting-state conditions.

In summary, we show that the high-frequency power-law exponent exhibits statistically significant spatial variability across brain regions, while in the low-frequency band the variability is within estimation error margins. These results support the view that scale-free dynamics may encode meaningful information about local circuit properties, but caution is warranted in extending these findings to functional or clinical interpretations without further data and investigations.

## 5. Conclusions

This study shows that high-frequency scale-free (1/*f*) slopes derived from stereotactic EEG recordings exhibit region-specific variability, even in the absence of external stimulation. While both low- and high-frequency ranges show 1/*f*-like behavior, only the high-frequency slope statistically differentiates between brain areas, suggesting its potential as a marker of local circuit characteristics. These findings support the idea that resting-state neural dynamics may carry regionally distinct signatures, aligning with earlier work on spectral fingerprints and scale-free activity.

Rather than introducing a novel perspective, our findings extend established frameworks—particularly those of [16,17]—to a large dataset of intracranial EEG, under controlled wakeful resting conditions. By applying power-law fitting techniques to physiologically normal brain regions, we contribute to the ongoing effort to characterize aperiodic neural dynamics as stable features of intrinsic brain organization.

While previous studies have linked 1/*f* slope to factors such as excitation/inhibition (E/I) balance and cognitive readiness [48,49,50], our dataset does not include behavioral, anatomical, or clinical correlates. Therefore, interpretations of the slope as a proxy for excitability or cognitive function should be made cautiously. Nonetheless, the observed regional differences in slope underscore the relevance of aperiodic spectral features for non-invasive functional parcellation of the brain.

This work supports the utility of 1/*f* spectral slope as a reproducible, interpretable, and spatially discriminative signal feature. Future studies combining aperiodic metrics with behavioral, developmental, or pathological variables will be crucial to clarify their functional and clinical significance.

## Data Availability

The original data presented in the study are openly available in the Montreal Neurological Institute (MNI) Intracerebral Recording Atlas at https://mni-open-ieegatlas.research.mcgill.ca/ (accessed on 15 January 2026). For the development of this work, no AI tools were used. The code used in this study is publicly available at: https://github.com/armonaite/PowerLawAnalysis.
